# Microbial metabolite facilitates virus control: inosine and T cells curb early-life influenza infection

**DOI:** 10.1038/s41392-025-02417-2

**Published:** 2025-09-23

**Authors:** Lisa Sevenich, Thi Trang Le, Lukas F. Mager

**Affiliations:** 1 M3 Research Center, Faculty of Medicine, University of Tübingen, Tübingen, Germany; 2 Department of Neurology and Interdisciplinary Neurooncology, Hertie Institute for Clinical Brain Research, University of Tübingen, Tübingen, Germany; 3 Department of Internal Medicine I, Faculty of Medicine, University of Tübingen, Tübingen, Germany

**Keywords:** Infection, Preclinical research

In a recent publication in *Cell*, Stevens et al. uncovered that microbiota disruptions reduced inosine levels and impaired CD8^+^ T cell immunity against influenza infection in early life. Treatment with inosine-producing bacteria or inosine alone restored T cell responsiveness through a nuclear factor interleukin 3 (NFIL3)- dependent mechanism.^1^

To model early-life dysbiosis, the authors pretreated pregnant dams with broad-spectrum antibiotics (ABX). Microbiota transfer from these mothers to their offspring was impaired, with notably reduced transmission of *Lactobacillaceae* and *Bifidobacteriaceae*. Infection of dysbiotic infant mice with influenza A (H1N1) revealed a diminished virus-specific CD8^+^ T cell effector response in the lungs and draining lymph nodes concomitant with lung damage and mortality. Moreover, rechallenge in recovered 8-week-old mice with H1N1 or the heterosubtypic, CD8^+^ T cell cross-reactive strain H3N2 further demonstrated an impaired viral-specific immune response later in life. This emphasizes the importance of perinatal microbiota-mediated immune priming to effectively protect against viral^1^- and bacterial pathogens.^2^ Still, ABX treatment during a critical developmental window may also directly impact T cell function and warrants further investigation.

This finding was strengthened by analyses of human lung tissue samples. T cells isolated from post-mortem human tissue of ABX-exposed infants showed impaired T cell differentiation and effector function compared to ABX-naïve infants. Indeed, a strong correlation between human and murine infants was detected in the CD8^+^ T cell transcriptome under dysbiotic conditions. Remarkably, influenza-specific T cell abundance, proliferation, and activation were greatly increased in CD8^+^ T cells from ABX-naive compared to ABX-exposed human tissues.

The observed microbiota-dependent immune functions may arise through altered microenvironment-, T cell intrinsic- or stochastic events occurring during infection. Thus, the authors isolated distinctly labeled, virus-specific CD8^+^ T cells from dysbiotic or control mice and transferred an equal mixture of CFSE-labeled cells into age-matched recipients. Following H1N1 infection, virus-specific CD8^+^ T cells from dysbiotic and control mice were then evaluated in the same recipient. CD8^+^ T cells from the dysbiotic donor proliferated less, showed reduced interferon (IFN)-γ and CD44 expression compared to the transferred control CD8^+^ T cells. Adoptive transfer of either dysbiotic or control CD8^+^ T cells into separate donor mice and consequent H1N1 infection revealed a drastic weight loss of recipient mice receiving dysbiotic CD8^+^ T cells. Together, this elegant approach identified a CD8^+^ T cell intrinsic mechanism as a driver of impaired antiviral immunity.

Single-cell RNA sequencing, pseudo time trajectory- and protein analysis highlighted a pronounced shift towards naïve CD8^+^ T cells in dysbiotic mice, whereas control mice were featured by an increase in memory T cells and exhausted T progenitor cells. Gene regulatory network analysis, as well as validation experiments on the protein level, identified NFIL3 as a central protein within a regulon that governs T cell effector differentiation and function. A T cell-specific NFIL3-deficient mouse model clearly demonstrated a similar impairment of T cell differentiation and viral defense as observed in dysbiotic mice. Analysis of histone tri-methylation linked NFIL3 to repressed transcription factor 7 (*Tcf7)* and lymphoid enhancer-binding factor 1 (*Lef1)* gene transcription, two well-known regulators of T cell proliferation and effector differentiation.

**Fig. 1:**
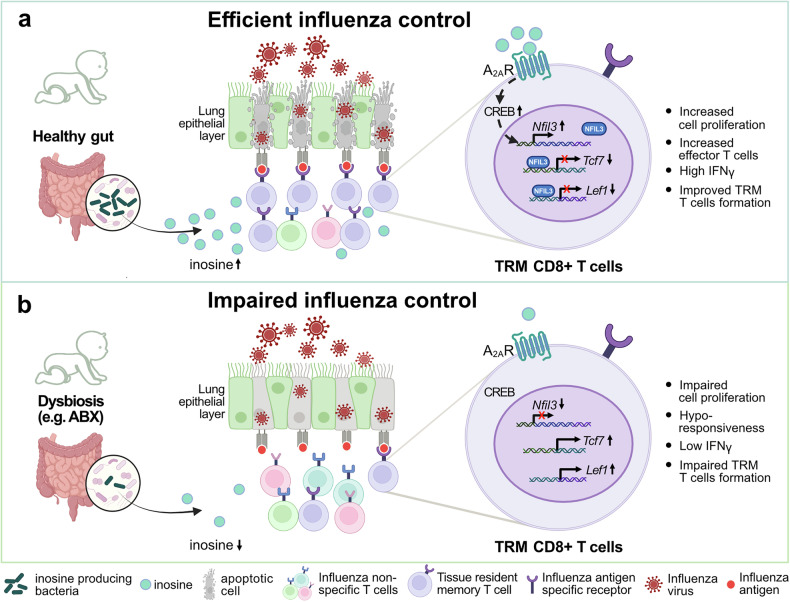
Bacterial-derived inosine induces NFIL3-T cell-dependent antiviral immunity in early life. Gut microbiome-derived inosine and consequent elevated systemic levels of inosine (e.g., plasma and lung) activate the adenosine 2 A receptors (A_2A_R) on CD8⁺ T cells. Receptor activation enhances cAMP response element-binding protein (CREB) activation and increases the expression of the transcription factor nuclear factor interleukin 3 (NFIL3). NFIL3 represses the transcription of transcription factor 7 (*Tcf7)* and lymphoid enhancer-binding factor 1 (*Lef1*). This promotes CD8⁺ T cell proliferation, effector differentiation, IFN-γ production, and supports the development of tissue resident memory (TRM) cells in the lung. Collectively, this strengthens antiviral immunity and protects newborns from influenza infection. **a** Depicting sufficient inosine abundance leading to efficient influenza virus control. **b** Depicting reduced inosine abundance, leading to impaired influenza virus control. Figure was created with BioRender, Affinity Designer 2 and Photoshop

One critical issue remained: How does the microbiome induce NFIL3-dependent CD8^+^ T cell viral response? Metagenomic sequencing from dysbiotic or control mouse and human fecal samples revealed *Haemophilus*, *Enterococcus*, *Lactobacillus*, and *Bifidobacterium* as significant discriminators between groups. Metagenomic-predicted metabolite analysis indicated inosine as a highly depleted metabolite in the dysbiotic microbiome state. The authors thus tested whether *Bifidobacterium pseudolongum* – a known inosine producer^3^ – or inosine treatment would improve T cell immunity related to influenza infection. *B*. *pseudolongum* monocolonization increased the T cell effector subset as observed in ABX- untreated and NFIL3-proficient mice. Inosine treatment in dysbiotic mice increased virus-specific CD8^+^ T cells, repressed *Tcf1* transcription, and protected from excessive weight loss following H1N1 infection. Comparison of human and mouse T cells from lungs of dysbiotic and control states linked inosine treatment to increased presence of NFIL3^+^ T cells, which was dependent on adenosine 2A (A_2A_) receptor signaling and induced phosphorylation of the downstream transcription factor cAMP response element-binding protein (CREB). Inosine also enhanced T cell proliferation and activation, thus unequivocally demonstrating a protective role of inosine treatment upon influenza infection (Fig. 1).

Stevens et al. add another facet to microbe-derived inosine as a potent immunomodulatory agent, in this particular case as an activator of CD8^+^ T cell-dependent antiviral immunity. Similarly, inosine has been highlighted as a potent adjuvant for checkpoint blockade and, more recently, in chimeric antigen receptors (CAR) T cell therapy in various cancer entities.^3,4^ We anticipate that future efforts will focus on how inosine may be used in the treatment of viral infections and other indications. Notably, the first patent for the use of inosine in cancer immunotherapy has been assigned (IMMUNOSPARKLE BIOSCIENCE LLC). Furthermore, inosine levels at the onset of viral infection or cancer may be valuable as a predictive- or patient stratification biomarker.

Some unresolved aspects of inosine remain mysterious. First, inosine treatment has previously been shown to repress pro-inflammatory immune function in various immune cells, including T cells.^5^ The availability of co-stimulator agents may control context-dependent effects and could underlie diverging observations.^3^ *Bifidobacterium pseudolongum*-derived inosine induces T cell activation against virus-infected and malignant cells.^1,3^ Thus, we anticipate future investigations to identify secondary microbe-derived factors that act in concert with inosine. Second, inosine pranobex (inosine combined with dimepranol acedoben), an antiviral agent, has been in use since 1971. Whether dimepranol acedoben acts as a secondary co-stimulatory agent for inosine to induce virus control, acts in a related or even distinct manner, warrants further investigation. Third, inosine alters the function of various immune cells, including CD4^+^ T and B cells that are crucial for virus control. We expect B cells, CD4^+^- and interactions with CD8^+^ T cells in the context of inosine treatment as another upcoming topic in the field.

Together, the study by Stevens et al. highlights a critical pathway by which *Bifidobacterium pseudolongum*-derived inosine promotes NFIL3-dependent CD8^+^ T cell immunity against influenza virus infection (Fig. 1). This work paves the way for future studies, including large patient cohorts probing the use of microbiome restoration strategies for high-risk infants —e.g., born preterm or exposed to antibiotics— and inosine-based therapies aimed at preventing severe disease progression.
